# A simplified approach to measuring national gender inequality

**DOI:** 10.1371/journal.pone.0205349

**Published:** 2019-01-03

**Authors:** Gijsbert Stoet, David C. Geary

**Affiliations:** 1 Department of Psychology, University of Essex, Colchester, United Kingdom; 2 Department of Psychological Sciences, University of Missouri, Columbia, Missouri, United States of America; University of Connecticut, UNITED STATES

## Abstract

The Global Gender Gap Index is one of the best-known measures of national gender inequality, used by both academics and policy makers. We argue that that this measure has a number of problems and introduce a simpler measure of national levels of gender inequality. Our proposed measure is based on sex differences in the opportunity to lead a long healthy and satisfied life that is grounded on educational opportunities. The measure better captures variation in gender inequality than other measures, with inclusion of outcomes that can be favorable or unfavorable to either sex, not simply unfavorable to women. We focus on some of the most basic measures available for 134 countries from 2012–2016 (i.e., disadvantages in children’s basic education, life satisfaction, and healthy life span) and we relate these to various measures, including the United Nations' Human Development Index. We found that low levels of human development are typically associated with disadvantages for girls and women, while medium and high levels of development are typically associated with disadvantages for boys and men. Countries with the highest levels of human development are closest to gender parity, albeit typically with a slight advantage for women. We argue that the disparities, when they are found, are related to the sexual division of labor (i.e., traditional gender roles) in poorly developed countries as well as the underinvestment in preventative health care in more developed nations.

## Introduction

The constructs "gender equality" and "gender inequality" are frequently used in both academic research (e.g., [1–2]), in the media, and by policy makers. Therefore, it is important that researchers and policy makers have reliable measures of these constructs. We discuss some of the challenges with existing measures and introduce a conceptual approach and an associated measure that helps to resolve or at least mitigate some of theses issues.

### Challenges for measuring gender inequality

Apart from political agendas, research on gender inequality has also almost exclusively focused on issues highlighted in the women's rights movement. Issues disadvantaging more men than women have been understudied (for a review, see [3]) and are not heavily weighted (if at all) in widely used measures of gender inequality, such as the highly cited Global Gender Gap Index (GGGI)[4]. Further, the GGGI truncates all values such that no country can, by definition, be more favorable for women than for men (for details see below). As a result, existing measures do not fully capture patterns of wellbeing and disadvantage at a national level. This is an important oversight, as there are issues that disproportionately affect boys and men. Among the many examples are harsher punishments for the same crimes [5] and an overrepresentation (93% worldwide) in the prison population [6]; compulsory military service (in living history or currently [3]); the large majority of homeless people without shelter are men [7]; higher levels of drug and alcohol abuse [8]; higher suicide rates [9]; more occupational deaths [10]; underperformance in schools [2]; and, men are more often victims of physical assault in general (see [3], p.30-33) and within schools, thus limiting educational opportunities [11]. Men are also overrepresented in occupations that are risky (e.g., exposure to toxins [12]) and physically taxing, such as front-line military duty, firefighting, mining, construction, or sewage cleaning.

In many countries, the retirement age is higher for men than women (although there are a few in which women's effective age of labor market exit is later, including Spain, Finland, and France), but even when it is equal, men often have fewer retirement years due to a shorter healthy life expectancy [13].

Finally, polygyny is tolerated in nearly half of all nations and is reported as being negative for women and it often is [14]; for a nuanced discussion see [15]. Polygyny, however, also means that more men than women in these nations are excluded from marriage, a family, and the opportunity to reproduce (given that polygyny leads to an unequal distribution of available partners). In other words, polygyny can be viewed as disadvantageous for most men (irrespective of the fact of whether it is disadvantageous for most women, as noted).

No existing composite measure of gender inequality fully captures the hardships that are disproportionately experienced by men and thus do not fully capture the extent to which any nation is promoting the wellbeing of all of its citizens. This is a major challenge, especially for the Global Gender Gap Index, which caps all disadvantages such that, by its definition, men can never be more disadvantaged than women. In other words, measures such as the GGGI will by design fail to measure any disadvantages experienced by boys and men.

### The Global Gender Gap Index (GGGI)

The GGGI [4], first published in 2006, is now one of the most established indices of national gender inequality across the world. All included nations are ranked based on four subindices, namely women’s 1) economic participation and opportunity, 2) educational attainment, 3) health and survival, and 4) political empowerment. These four subindices are each based on several variables, each weighted differently. The scores for each subindex range, theoretically, from 0 to 1, whereby 1 indicates that women have parity (or that men fall behind, given that values higher than 1 are capped).

There are several difficulties with the way the GGGI is composed. For one, there is no defensible rationale for truncating scores on an ‘equality’ measure when they disadvantage boys or men. Further, certain subindices may result more from choice than from a disadvantage. For example, fewer young adult men than women enroll in tertiary education in most developed nations. Although this may represent a disadvantage for men, it may also simply reflect a preference for a less academically oriented pathway into vocational occupations [16]. Another example is the earnings gap between men and women, which may well reflect a strategic and desired division of labor within families, rather than a disadvantage to women [17]. This may similarly affect the desire to engage in high-level politics, which require exceptional demands on the work-life balance, and which therefore may be less desirable to many woman [18]. Altogether, these differences in occupational preferences and strategic divisions of labor in family life may skew quantifications of true gender inequality. We are not arguing here that the GGGI is definitely skewed because of this, but merely that there is currently no way of knowing whether inequalities in outcomes are the result of inequalities of opportunities; therefore, not using these variables may resolve this potential skew in the GGGI.

The weighting of GGGI subindices is another issue, as is the degree to which the chosen variables are relevant for the majority of the population. More specifically, the subindex "health and survival" is the combination of the sex ratio at birth (which may indicate sex-specific abortions) and healthy life expectancy. The underlying argument is that sex-specific abortions of girls indicate a negative attitude toward women. This may in fact be the case (but for a philosophical argument see [19]), but is a very indirect measure, and not a good indicator of the health and survival of living persons. We believe that weighing this much heavier (weight 0.693) than healthy life expectancy (weight 0.307) undervalues the health and survival of actually living persons. Given that men typically have a shorter life expectancy, this again skews the GGGI toward overestimating female disadvantage. At the very least, if birth ratios are considered, they should be an independent index.

### A simpler way of measuring gender inequality

Given the myriad ways in which women (e.g., childhood marriage) and men (e.g., occupational hazards) can be disproportionately disadvantaged in any given nation, it is practically impossible to reach consensus on how to measure and weight all of them. Even if there could be a consensus about exactly which of the many theoretically possible variables should be used to express gender inequality, there is a practical limitation; there would be few countries for which all variables could be reliably measured, thus resulting in an index that is not truly global. An index that captured core aspects of life that are common to all people and can be measured with a few readily available indices would help to address these issues.

We propose that these core aspects of life are reasonably well captured by people’s opportunity to live a long and healthy satisfied life that is grounded on educational opportunities in childhood. Accordingly, our Basic Index of Gender Inequality (BIGI) is the ratio of women to men on three core dimensions of life; 1) Educational opportunities in childhood; 2) Healthy life expectancy (the number of years one can expect to live in good health); and, 3) Overall life satisfaction. We believe these 3 components complement each other in important ways; leaving one out misses an important aspect of what defines a good life. For example, a person may have a satisfied and long life, but without educational opportunities, such a person might not have had a chance to develop his or her talents. Or, a person may have a satisfied life following a good education, but dies prematurely. And finally, one may be educated and live long, but without much life satisfaction. We believe that the three components together capture the core of what defines a healthy and long satisfied life that is grounded on equal educational opportunities. We believe that these are the minimal components needed for living a fulfilled life, and that our indicators indirectly reflect other aspects of life (e.g., a decent standard of living, which will be reflected in healthy life span and life satisfaction).

The use of overall life satisfaction is a key feature of BIGI. The idea is that while it is very difficult to determine the degree to which men and women are disadvantaged in any particular aspect of life, an overall assessment of life satisfaction likely reflects the combination of advantages and disadvantages they have experienced, whatever they might be [20].

### A case for an additional measure of gender equality

Working with our definition and 3 main components has a number of advantages over commonly used gender inequality measures, or at the very least provides one additional way of assessing inequality. First, we work with a clear overarching concept, namely a healthy life grounded in educational opportunity. In contrast, the highly cited GGGI lacks an overarching concept as argued, for example, by Hawken and Munck [21]. Second, our definition has few components, which reduces the potential for the selection bias and weighting problem common with some other measures. Third, because of its simplicity, the data are available for a large number of countries (i.e., the more separate issues one would include, the smaller the number of countries for which a complete set of data is available).

The BIGI focuses on aspects of life that are directly relevant to all people and avoids the difficulties of choosing and weighing indices that are relevant in some contexts but not others, and often may reflect life choices rather than restricted opportunities (e.g., the ratio of male to female national politicians is only relevant to the tiny proportion of people who choose a political career, see below).

As noted, the BIGI is thus simpler than the GGGI in regard to components and weighing of components. An important and intended side effect of the built-in simplicity in calculation and focus on well-being is that the BIGI is less focused on women’s interests and more focused on gender equality. We are not, however, arguing that the BIGI should replace the GGGI. In fact, we have used the GGGI in our own research and believe that it is serves a useful function; for example, it is a useful measure of women’s emancipation, such as political and financial participation.

In addition to the GGGI, we believe that there is a need for a measure that captures a more basic–the underlying dimensions are understandable to and reflect the wellbeing of the typical citizen in any country–sense of gender equality. Or stated differently, a measure that is less influenced by many complex assumptions about men and women’s motivations and aspirations. For example, it is an assumption that the underrepresentation of women in national politics is a reflection of gender inequality. After all, it may simply reflect a sex difference in occupational choices [22]. For example, as noted, it is now reasonably well known that men and women have different preferences in the work/life balance [18].

To avoid the complexity of assumptions around behavioral drivers, the BIGI does not include measures that might be due to socio-political emancipation in some contexts, or by personal and reasonable choice in other contexts (e.g., a desire to become politician, a desire to participate in tertiary education, or the desire to earn a wage). After all, one can, in principle, live a good life without earning a wage (as long as one is part of a supporting family) and one can live a good life without earning a college degree. Apart from this, decisions not to earn a wage or a degree, or decisions to follow traditional gender roles can be part of cultural beliefs one feels strongly attached to and happy with.

We agree that one might be seriously concerned about the fact that more men choose to earn a wage than women, and that more women enroll at university than men. And we agree that one might be seriously concerned about the fact that certain cultural or religious beliefs can lead to a rigid gender-specific division of labor. At the same time, though, we realize that any such concerns are the result of our own social, cultural, and political bias. In a different time and place, one might have very different biases. One of the specific features of the BIGI is that it aims to be unbiased, that is, we do not need to make assumptions about underlying personal motives or about cultural, political or religious strictures to obtain a broad assessment of how well a typical citizen in any given county can obtain a basic education and live a long and satisfying life. We are not claiming that this is necessarily better than the GGGI, but we believe that there are research projects and policy issues in which such a measure can add value to currently used measures, including the GGGI.

## Materials and methods

### Data sources

For the calculation of BIGI, we used two data sources covering the 5-year period 2012–2016, inclusive. From the Global Gender Gap Reports [4], we used the data on healthy life span, enrolment in primary and secondary education, and the literacy rate. From the Gallup World Poll (www.gallup.com), we used the question "Life Today", measuring overall life satisfaction (details below). These data are available for 134 nations (S1 Table).

The Gallup World Poll Question "Life Today" is formulated as follows: "Please imagine a ladder with steps numbered from 0 at the bottom to 10 at the top. Suppose we say that the top of the ladder represents the best possible life for you, and the bottom of the ladder represents the worst possible life for you. On which step of the ladder would you say you personally feel you stand at this time, assuming that the higher the step the better you feel about your life, and the lower the step the worse you feel about it? Which step comes closest to the way you feel?"

In this article, we also report the Human Development Index [23], the Gender-related Development Index (GDI, [23]) and the Global Gender Gap Index [4] for comparison. For HDI and GDI, we used the data 2012–2015. The HDI is composed of three components, namely life expectancy at birth, years of schooling, and gross national income per capita. The GDI is a gender-specific version of the HDI.

For comparison of BIGI with health-related behavior, we used alcohol consumption data from Wilsnack and colleagues [24] and the World Health Organization (WHO) for the year 2016, which are available in Global Health Observatory (GHO) data sets (http://apps.who.int/gho/data/node.wrapper.imr?x-id=4402). From the same WHO observatory, we also included maternal mortality (http://apps.who.int/gho/data/node.main.WOMENSDG31?lang=en) and overweightness (Body Mass Index > 25, http://apps.who.int/gho/data/node.main.A897A?lang=en).

For determining the population sizes of countries, we used the average population size of countries for the years 2012–2015, inclusive, as reported in the Human Development Report [23].

### Calculation

We calculated the BIGI score using the following steps. For each country, we calculated the ratio of women to men for healthy life span and for overall life satisfaction. Thus, men and women scoring equally results in a value of 1, women scoring lower than men in a value below 1, and women scoring higher than men in a value above 1. For children’s education, we performed a more complicated calculation. First, we calculated three education ratios, namely for primary education enrollment (i.e., ratio of girls to boys enrolled), for secondary education enrollment, and for literacy rates. Of these three ratios, we took for each country the value that deviated most from 1, that is, from parity. In this way, we ensured that the lack of opportunity in any aspect of education is not obscured by the other education indicators. For example, a country in which the ratio of literate females to males is 0.7, the ratio in primary school enrollment is 0.8, and the ratio secondary education is only 0.5, we would take the 0.5 value to express the level of educational gender inequality in that country. (Note that rarely, not all three educational variables were available; in those cases, we ignored the missing data and choose the available value that deviated most from parity).

Taking the most extreme disadvantage in children’s education (of literacy rate, primary school enrollment, and secondary school enrollment) provides a more sensitive indicator of disparities than does averaging the three. This seems justified, because illiteracy, for instance, can potentially be a better indication of basic educational opportunities than enrollment in primary or secondary education (e.g., children might be officially enrolled but rarely show up); this is confirmed by the fact that the gender gap in primary education enrollment does not correlate strongly with the gender gap in the literacy rate (i.e., for 2016, Spearman's rho, r_s_ = .20, for 2015, r_s_ = .30), as one would expect if primary education enrollment in and of itself was not sufficient to learn how to read. Next, we calculated for each country the average of the three ratios (i.e., healthy life span, educational opportunities, and overall life satisfaction). In order to have 0 representing parity, we subtracted the resulting average from 1. As a result, BIGI values below 0 represent a disadvantage for boys and men, while values above 0 a disadvantage for girls and women (S1 Table).

Finally, because there might be small fluctuations from year to year due to "noise", we decided to collate the data of a 5-year period (2012–2016).

Note that for calculating the ratio of women to men, we adjusted the ratios to ensure symmetry, as is commonly done ([25], p.3). The adjustment prevents overestimation of disadvantages for men. For example, a rate of 0.8 for women and 0.9 for men (sex ratio 0.8/0.9 = 0.89) differs 0.11 from 1, whereas a rate of 0.9 for women and 0.8 for men (ratio 0.9/0.8 = 1.13) differs 0.13 from 1. The adjusted ratio (dividing the smaller by the larger value) differs 0.11 from 1 irrespective of gender.

**Fig 1:**
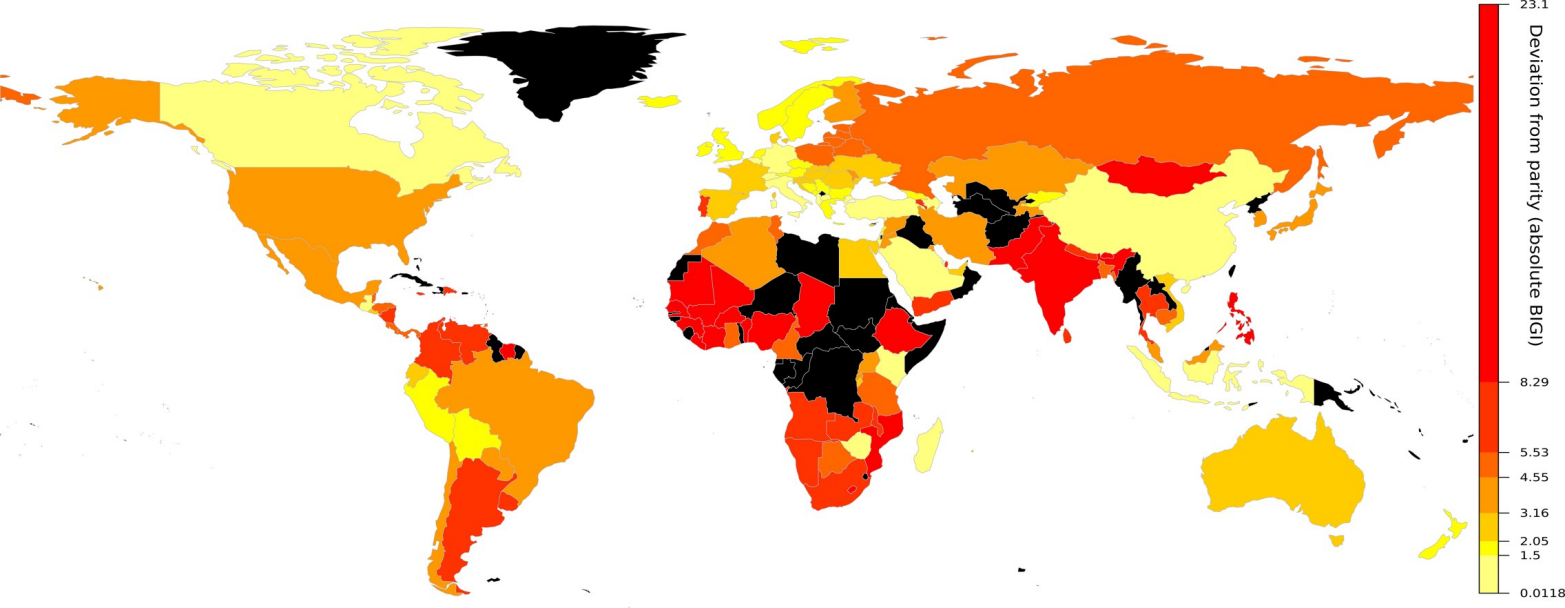
Levels of deviation from gender parity based on the absolute Basic Index of Gender Inequality (BIGI) scores for 134 nations (representing 6.8 billion people). Black indicates countries without data.

All data were processed with the statistical software R [26]. The world map (Fig 1) was prepared using the R package “rworldmap” [27].

### Example of calculation

We use the United States to illustrate the calculation of the BIGI scores (S1 Table). We start with the BIGI 2016. Note, all ratios in the calculation below are adjusted as described above (i.e., smaller value divided by larger value).

1. The healthy life span ratio for women (71 years) to men (68 years) in the US in 2016 is 1.0423 ([4], p.55).
2. For 2016, enrollment sex ratio in primary education and literacy are listed as 1.00 ([4], p.50-51), whereas the sex ratio in secondary education enrollment ([4], p.52) as 1.0326, and literacy rate as 1.00. For the education component of BIGI we take the value that deviates most from parity, in this case 1.0326.
3. From the Gallup World Poll data 2016, we took the ratio of women’s life satisfaction in the USA (6.9094) and men’s life satisfaction (6.6947), which was 1.0311.
4. The average of the above (healthy life span ratio, 1.0423, education score, 1.0326, and the life satisfaction score, 1.0311) is 1.0353.
5. In order to have 0 as representing parity, we subtracted the results from 1, that is 1–1.0353 = -0.0353. In other words, in the US in 2016, the BIGI deviation from parity was 3.5% (in favor of women, because the value is below zero).
6. We calculated the US’s BIGI scores for the years 2012–2016 the same way (-0.0357, -0.0419, -0.0246, -0.0271,-0.0353, respectively), resulting in the BIGI average for 2012–2016 of -0.0329.

### Ethical approval

No institutional ethical approval was necessary for carrying out this secondary data analysis of publicly available datasets.

## Results

### BIGI scores

Using the calculations described above, we created BIGI scores for 134 nations (representing 6.8 billion people in the studied period). As noted, BIGI scores below 0 mean that men are more disadvantaged than women, and scores greater than 0 mean that women are more disadvantaged than men (S1 Table).

In 91 (68%) of the 131 countries, men were on average more disadvantaged than women, and in the other 43 (32%) countries, women were more disadvantaged than men. The international median of the BIGI is -0.017 (SD = 0.062), that is, nearly a two percent deviation from parity, favoring women. Examples of a (roughly) 2 percent deviation from parity include a healthy life span of 66 for women and 65 for men (e.g., in Saudi Arabia in 2016), 96% of girls vs. 94% of boys enrolled in secondary education (e.g., in Cyprus, 2016), or a 5.5 vs. 5.4 score on the life satisfaction scale (e.g., in Portugal, 2016).

**Fig 2:**
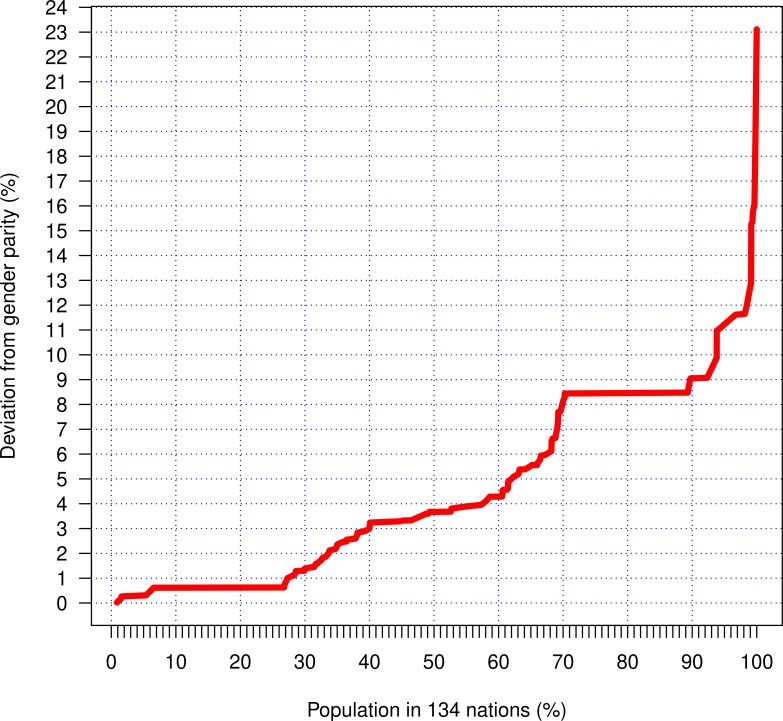
Cumulative deviation from gender parity (y-axis) related to the cumulative distribution of world population (6.8 billion people in 134 nations). This shows, for example, that 50% of the studied population lives in a nation with less than 4% deviation from gender parity.

The international median for the absolute (i.e., unsigned) value of the BIGI deviation from parity is 0.036, indicating a 3.6 percent deviation from parity, independent of which sex is disadvantaged. Thirteen countries had less than a 1 percent deviation from parity overall, but all had larger deviations for the individual components of the BIGI (see below). Ninety countries had less than a 5 percent deviation from parity overall, again typically with larger differences for specific components of the BIGI (Fig 1). This corresponds to around 62% of the studied population (Fig 2).

As mentioned, the deviations from parity in each of the three individual components of the BIGI can be larger than the BIGI itself. This means that a country with an overall apparent lack of gender inequality may have one sex falling largely behind in one component of the BIGI (e.g., life satisfaction) while the other sex falls behind largely in another facet (e.g., education). This means that despite overall parity, the country still has much work to do to achieve parity in individual components of the BIGI. We therefore argue that it is important to consider both the overall level of parity and the level of parity (or lack thereof) in each of the individual components.

Take for example Saudi Arabia. It has a very low overall deviation from parity (0.15%) but relatively high disparity in individual components (e.g., a 7% disadvantage for women in education and a 5% disadvantage for men in life satisfaction). In other words, because men's and women's disadvantages average one another out, it reaches a high level of overall parity. Indeed, Saudi Arabia is a country with one of the largest differences between its overall level of parity and the lack of parity in the individual components (S1 Table).

### BIGI and human development

**Fig 3:**
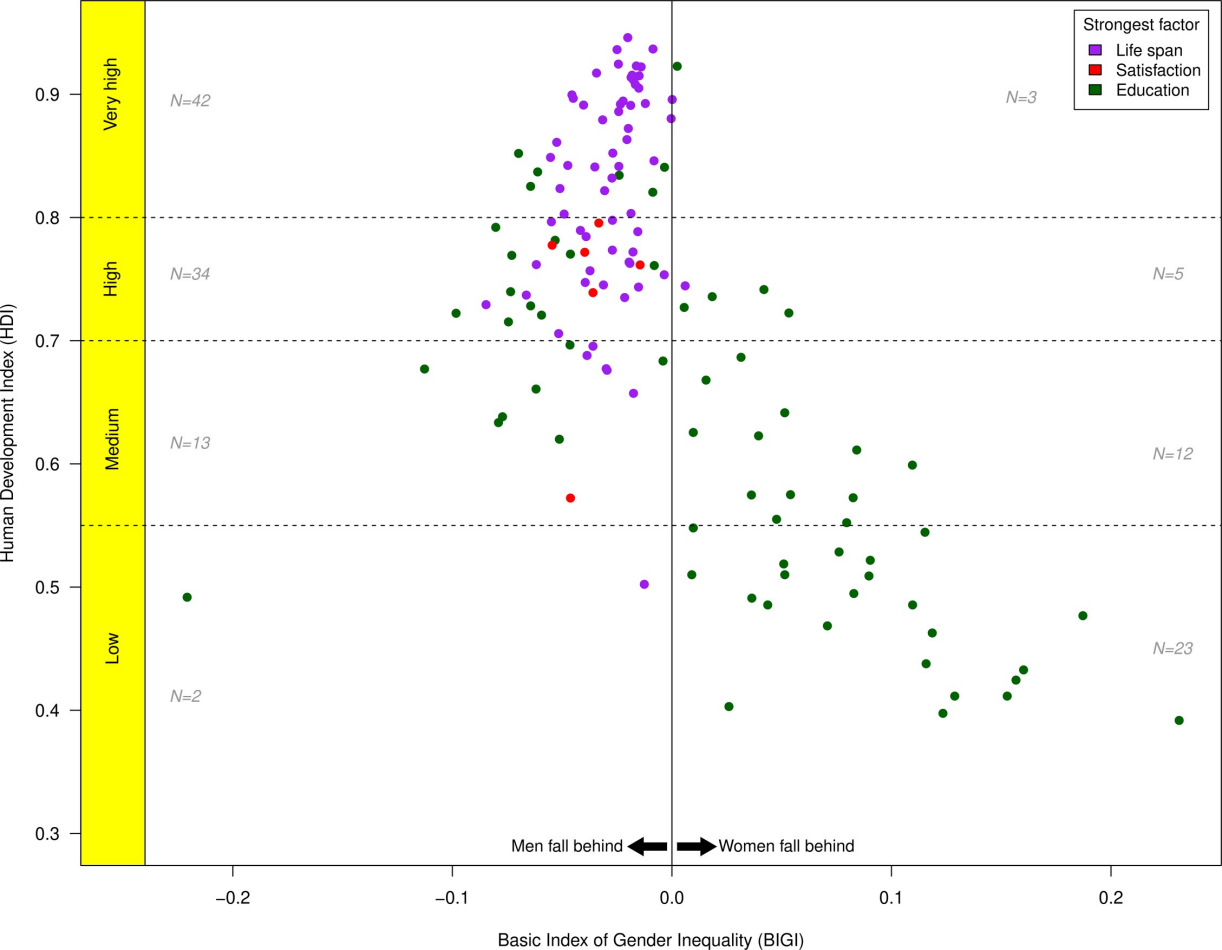
The Basic Index of Gender Inequality (BIGI, x-axis) as a function of the Human Development Index (HDI, y-axis). BIGI is the average of 3 components: Ratio in healthy life span, ratio in overall life satisfaction, and ratio in educational opportunities during childhood (see Materials and Methods for details). Deviation from zero implies the extent of gender inequality. The plot shows the largest contributor to the overall score for each nation: Purple dots indicate healthy life span is the most important component, green dots indicate educational opportunities, and red dots indicate overall life satisfaction. The Ns indicate for each level of HDI how many nations have a BIGI score greater than 0, and how many less than 0.

We compared the BIGI score with the Human Development Index (Fig 3). A visual inspection of the figure reveals that women and girls fall behind most in the least developed nations, whereas there is a narrowing of the spread in values among the very highly developed nations. Nations at medium levels of development vary the most, with some having the greatest disadvantages for women and girls, while others having the greatest disadvantages for men. It is also clear that education is most often the reason for why women and girls fall behind, whereas men’s disadvantage is largely in a shorter healthy life span in the high and very highly developed nations.

**Fig 4:**
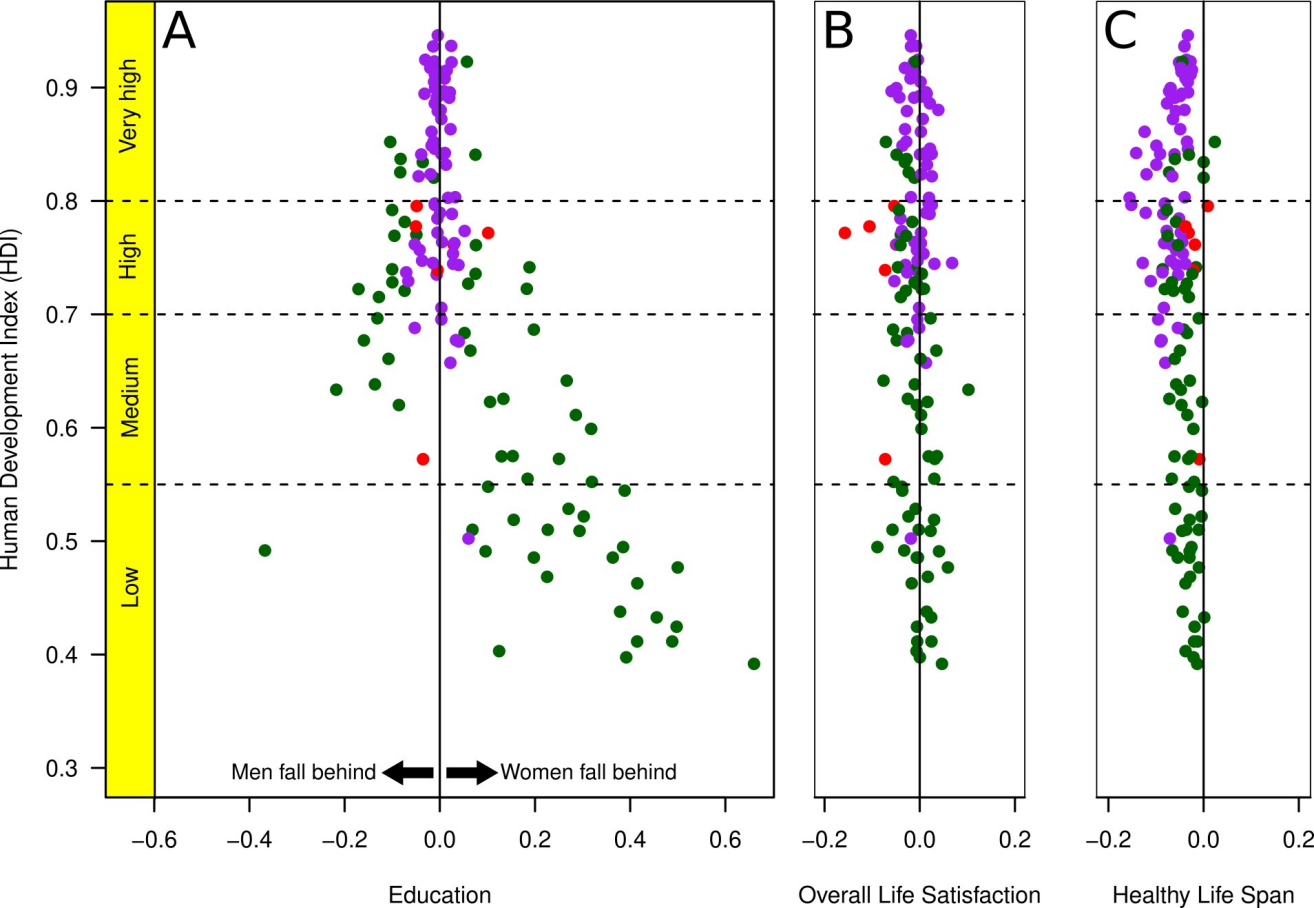
Gaps in the three main components of the BIGI as a function of human development (y-axis). The color coding is as in Fig 3. A: Education gap (note that the outlier point in the low HDI range is Lesotho). B: Overall life satisfaction gap. C: Healthy life span gap. As in Fig 3, negative values indicate disadvantages of men, and positive values disadvantages of women.

In order to further illustrate the contributions of the three components of the BIGI, we plotted each of them in relation to HDI (Fig 4), but keeping the color coding (of Fig 1) for the most important component for each country. The largest variability is found in the education component, and women’s healthy-life span is longer than that of men in nearly all countries. The only exceptions for the latter are the United Arab Emirates, Bahrain, Kuwait, Mali, and Qatar. In the latter 3, the healthy life span of men was slightly longer than that of women (S1 Table).

**Fig 5:**
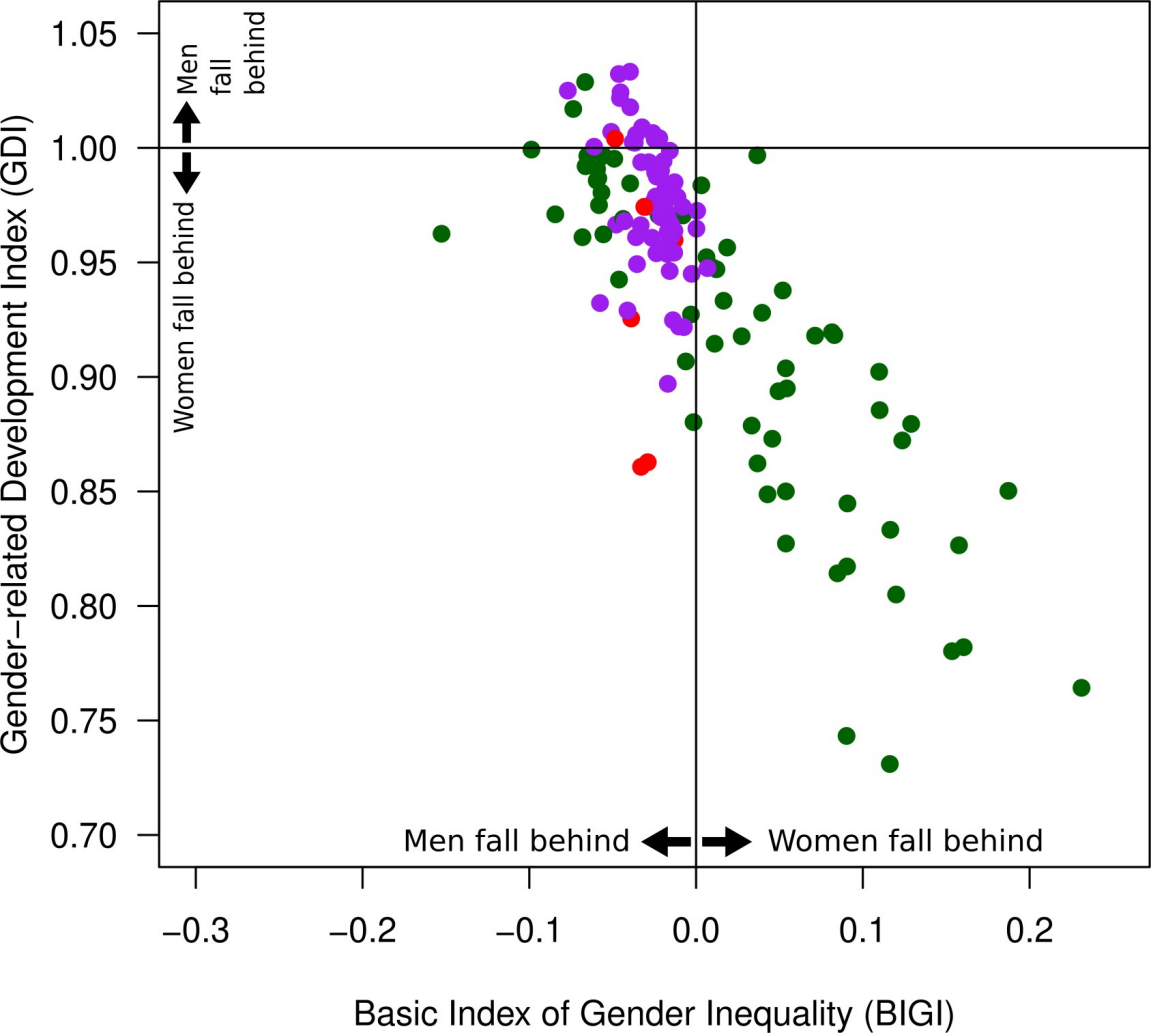
Relation between the Basic Index of Gender Inequality (BIGI) and the Gender-related Development Index (GDI). The GDI is the ratio of the HDI for women to HDI for men. Color coding as in Fig 3.

The Gender-related Development Index (GDI, Fig 5) is essentially a ratio of women to men in relation to the HDI index. We found a strong relation between this index (GDI) and the BIGI (r_s_ = -.74, p < .001, n = 132). An important difference is that the BIGI is more sensitive to men’s disadvantages than the GDI; the BIGI reveals more countries in which women have an advantage over men (68%) than the GDI (13%). Moreover, the GDI and BIGI show similar levels of absolute parity, with 61% of countries in GDI showing less than 5 percent deviation from parity and 67% of countries in BIGI. In contrast, the smallest deviation from parity in any country using the GGGI is 13%.

### Using BIGI to understand sex differences in culturally dependent outcome variables

We investigated the potential applicability of BIGI for understanding issues that may or may not be related to measures of gender inequality. We selected a variety of measures that are within the control of a society's policy makers. Our choice of examples was influenced by the finding that the health-related BIGI component plays a relatively large role in very highly developed nations. These nations are of interest, because their populations have a high standard of living; for them, one way to make a meaningful improvement is to reduce gender inequality (whereas for less developed countries, there may be more urgent priorities).

Unhealthy lifestyle choices related to alcohol, tobacco, exercise, and unhealthy food will reduce the health status of populations. The success of approaches to preventing unhealthy lifestyle choices may depend on gender (and so contribute to the level of gender inequality).

**Fig 6:**
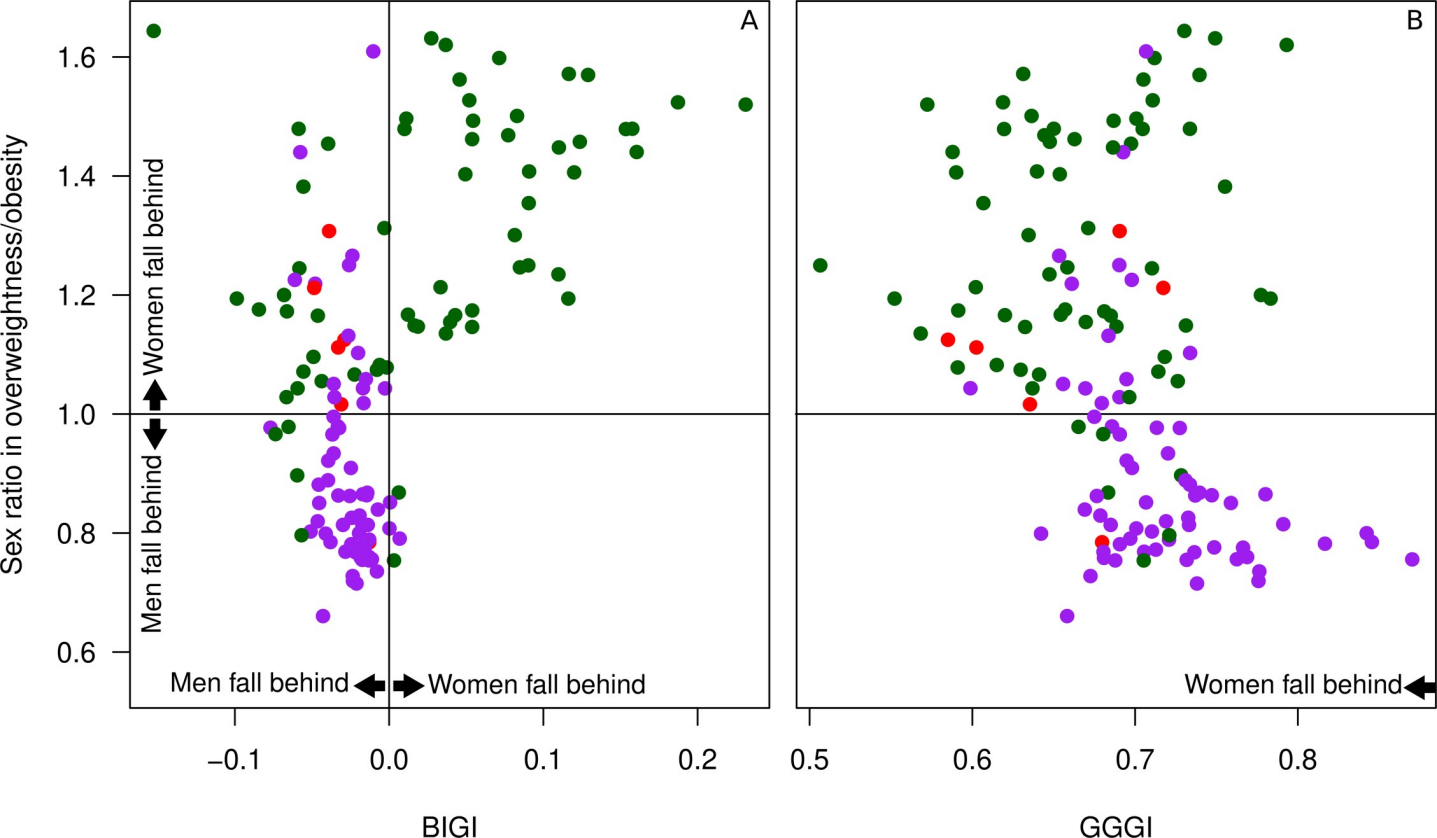
The sex ratio in overweight and obesity (BME > 25, y-axis), with numbers below 1 showing that fewer women than men are overweight/obese. Color coding as in Fig 3. A: BIGI scores and the sex ratio in overweightness/obesity. B: GGGI scores and the sex ratio in overweightness/obesity.

Our first example of a health-related lifestyle factor is being overweight (body mass index > 25) or obese (body mass index > 30). Worldwide, obesity is three times more likely to result in premature death than malnutrition [28]. Importantly, the sex ratio of being overweight varies strongly between countries, ranging from 0.66 in Japan (28% of men vs 19% of women with a BMI over 25) to 1.64 in Lesotho (18% of men vs 51% of women). Of interest is that a higher proportion of overweight men is almost uniquely observed in countries where BIGI favors women (Fig 6A). Countries in which women are more disadvantaged than men (BIGI > 0) all show a higher proportion of overweight women. Therefore, we believe that BIGI offers a different and potentially more useful insight into this gender-specific disadvantage than the commonly used GGGI (Fig 6B).

**Fig 7:**
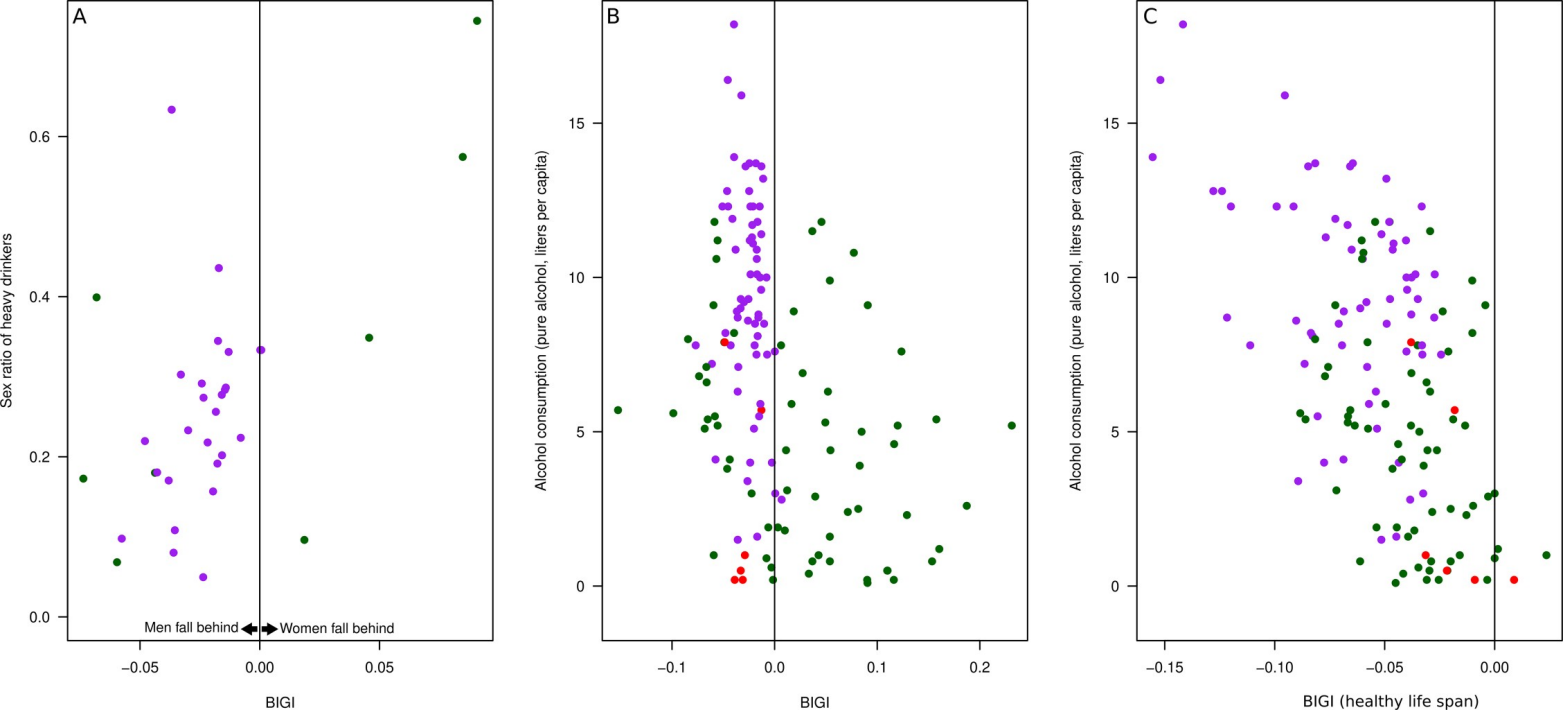
The relation between BIGI and alcohol consumption. Color coding as in **Fig 3**. A: BIGI and the gender ratio among heavy drinkers. B: BIGI and general alcohol consumption. C: The healthy life span component of BIGI and general alcohol consumption.

Alcohol consumption is our second example of a health-related factor (Fig 7). As is the case with being overweight, alcohol consumption is a preventable cause of poor health. There are only limited data sets available for sex differences in alcohol consumption. For example, Wilsnack and colleagues [24] provide data for 36 nations, 34 of which have BIGI scores (Fig 7A). Most of these countries have a BIGI score below 0 (i.e., disadvantaging men more than women) and a higher level of development (HDI, median 0.89) than the mean of all 134 nations with BIGI scores. In these countries, the average sex ratio in regard to high-volume drinkers was 0.26, that is, there were on average 4 times more male than female high-volume drinkers (i.e., > 8486 grams in 12 months), and this was related to BIGI (r_s_ = .47, n = 34).

The Wilsnack data set [24] contains only a quarter of all nations with BIGI scores. To include more nations, we used the WHO data on alcohol consumption, which is not gender-specific. However, based on Wilsnack's data set [24], it is reasonable to assume that men's overconsumption is related to the total amount consumed. Therefore, we assumed that the WHO data on alcohol consumption of the whole population may be an indicator of men's greater alcohol consumption (Fig 7B). We compared alcohol consumption per capita with the gender gap in healthy lifespan and found that countries with higher levels of consumption have a larger gap in healthy life span (Fig 7C, r_s_ = -.53, p < .001, n = 134).

**Fig 8:**
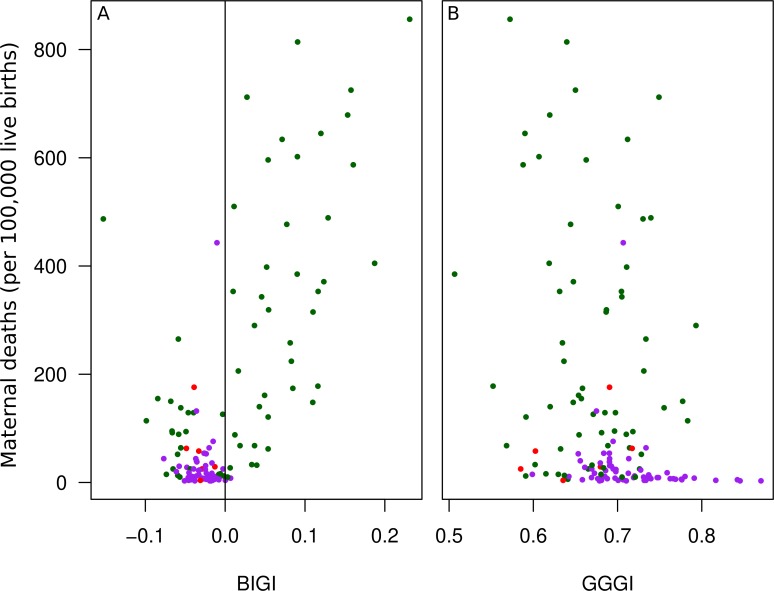
**Maternal death during live births rate as a function of BIGI (A) and GGGI (B).** Color coding as in Fig 3.

Our third example is the mortality of mothers during live births. As expected, the rate of maternal deaths during live births is clearly higher in countries where women are more disadvantaged than men (Fig 8A). Again, BIGI (Fig 8A) offers more of an insight than the GGGI (Fig 8B).

## Discussion

We introduced a novel and simplified way of tracking gender inequality that focuses on measures of basic well being (healthy lifespan, pre-tertiary education, and life satisfaction) and that avoids measures based on more complex social, political, or cultural assumptions or biases. As with other composite measures, our BIGI scale reveals considerable differences in national levels of gender inequality that in turn are related to levels of human development (Fig 3). Countries with very high levels of human development are closest to gender parity, and deviations from this typically favor women. This disparity in women's favor is also common in countries with high (but not the highest) levels of human development. The picture is more mixed among countries that are intermediate in human development; there are nearly the same number of countries where women fall behind as countries where men fall behind, and with a larger spread of values. It is in the least developed countries where women nearly always fall behind men on our measure.

An explanation for this non-linear relation between gender inequality and human development might be related to three factors. First, the largest variability among countries is due to sex differences in educational opportunities, and centered largely in less developed nations. It may be that investment in education is an economic luxury in these contexts, especially investment in girls' education in contexts where people follow an agricultural lifestyle with traditional gender roles. A good example of such a country is Chad, one of the poorest countries in our survey and with a harsh climate and with an economy mostly based on livestock herding [29]. Although this is a common pattern in sub-Saharan Africa, there are exceptions. For example, Lesotho has the one of the largest gender gaps in education in favor of girls, which appears to be due to boy's involvement in herding and other low-skilled opportunities in the labor market [30].

The source of variability in overall life satisfaction and healthy life span are nearly identical. Of interest is that women have a longer healthy life span than men in nearly all countries. Although this is a pattern common to mammals [31], the cross-national variation cannot be simply due to biological sex differences alone. This is because it is exaggerated in certain regions of the world (e.g., eastern Europe, [32]) that are likely related, in part, to behaviors (e.g., alcohol and drug abuse) resulting from wider cultural and economic challenges (although these are not well understood, [33]). Given the general sex difference in life span, it is noteworthy that there are a few countries (United Arab Emirates, Bahrain, Kuwait, Qatar, and Mali) in which women's healthy life expectancy was equal to or shorter than that of men. These nations all share the same majority religion (Islam) and have a desert climate, but the factors that produce this reversal of the general pattern for life span cannot be determined from our data with certainty (we speculate that low levels of alcohol consumption in Islamic countries may play a role).

It should also be noted that there is a strong linear relation between the BIGI and the gender-related index of development, the GDI. A crucially important difference between the BIGI and the GDI is that the latter takes the gender earnings gap into account. While financial resources are related to wellbeing, we argue that individual earnings of men and women are not necessarily a good indicator of financial resources, given that most adults live in households where income is shared [34]. In fact, one may argue that in many contexts the division of labor and income in families can provide advantages to women, giving them an opportunity to benefit from the family income to cover costs associated with raising children (if they wish to do so), as can be found in nations with high levels of human development (e.g., [17]).

Some readers may be surprised that Saudi Arabia, frequently portrayed as unfair to women in the media [35,36], has a relatively high level of overall average gender parity. This seems contradictory, because much has been reported about restrictions on women's rights in Saudi Arabia (e.g., the Saudi government only recently changed its policy on women driving a car), while Northern European countries have a reputation for progressiveness (including female participation in national politics). Apart from other academic research showing that "Islam, democracy and gender equality are not inherently incompatible" ([37], p. 518), three critically important points should be noted here.

The first is that the lack of gender inequality does not imply that women or men have abundant opportunities in life (see Introduction), and neither does it mean that a country is free of sexist attitudes; all that matters for the expression of gender parity using the BIGI (or any other composite measure of gender inequality) is whether there are overall differences in disadvantages between the sexes. Indeed, Saudi Arabia has much to do, because girls fall behind considerably in educational opportunities (7%, S1 Table), while men fall behind in both healthy life span and life satisfaction.

It is exactly because of this potential of having a low level of average overall gender inequality and still have relatively large disparities in the individual components that we have also provided a score for the average absolute deviation from parity (AADP); this better reflects the amount of work to be done (in a society) to resolve all relevant gender disparities as captured by the BIGI. We have ranked the BIGI according to this latter score (S1 Table). It should be noted that our approach of ranking in accordance to the average absolute deviation from parity is a novelty in gender equality indicators. It is another dimension that should be taken into account when comparing nations; it is particularly useful to prevent overestimation of progress in countries in which both men and women fall behind in different aspects of life (such as in Saudi Arabia).

Second, as argued in the introduction, the general focus in the area of gender inequality is often on issues relevant to women, while discounting men's issues. For example, while the issue of Saudi women not being allowed to drive has received much media attention, little is reported about issues affecting Saudi men. Little is written, for example, about the challenges many lower-status men have in finding a partner in a country where polygyny is legally practiced, yet this almost certainly undermines their health and wellbeing, as well as their life satisfaction. Moreover, although girls fall behind in educational opportunities (e.g., years schooling), the girls that attend school outperform boys by a larger degree than in many other countries [38], raising questions about Saudi education for boys as well. In this context, we would like to note that the often-touted bias toward the interests of men applies to high status men, not men in general.

Third, differences in cultural and religious views may influence one’s assessment of advantages and disadvantages in life. For example, most people in Saudi Arabia subscribe to a set of societal rules that may be difficult to understand from a Western point of view and may well be seen as a disadvantage by non-Muslims. Common examples are extended periods of fasting, dietary rules, and dress code, all of which are considered as a discomfort to non-believers, but are an integral part of social life in this context. In short, disadvantages cannot always be defined objectively. The overall life satisfaction score is culturally independent, and therefore may be a better broad measure of advantages and disadvantages that people experience in their lives than a composite of variables (e.g., wage gap) that may be more relevant in some contexts than others.

It should be noted that of the three components, education shows the most variability across nations, and is the factor that can be most directly influenced by governments. It is also a factor that is recognized as influencing one’s opportunities in life and is seen as a valuable resource in most places in the world [39].

Once a certain level of economic development is achieved, there is no rationale for why girls or boys should be disadvantaged in access to and availability of primary or secondary education. This remains a challenge in many parts of the world [25]. Across the nations assessed here, there are 76 countries in which girls are educationally disadvantaged compared to 58 countries in which boys are disadvantaged. The largest gaps occur in the least developed nations, and reflect substantive disadvantages for girls, as reported elsewhere [39]. African countries play a major role in this, followed by South Asian countries. Economic advances in these regions that support expanded educational opportunities will likely result in relatively large educational gains for girls and women.

### Limitations

One notable limitation of BIGI is that we cannot determine statistical significance of country comparisons. In other words, we cannot determine if there is a statistically meaningful difference between the values of, say, Bahrain and Great Britain (ranked numbers 1 and 2, S1 Table). It is important to point out that the same limitation applies to all other cross-national indicators of gender inequality we are aware of. This limitation is due to a lack of information about variability in the measures used. We do not believe that this limitation is all encompassing. In fact, this limitation has not kept researchers from using the popular Global Gender Gap Index.

### Conclusions

Our simplified measure of gender inequality tracks well with national variation in human development, speaking to its validity and utility, and seems to provide a more nuanced picture of inequality than commonly used measures, such as the Global Gender Gap Index (GGGI). We are not necessarily arguing that measures such as the GGGI should not be used, but rather inclusion of the BIGI in such studies will provide additional and different information and in doing so will provide a more complete assessment of gender equality. Our overall results suggest that in today's world, many countries have achieved an historical level of gender parity. Even so, resolving gender inequality is only part of what is needed to ensure that all people can reach their full potential; overall gender parity on its own is not sufficient, because it can simply mean that both men and women lack opportunities in different facets of life quality.

Internationally, improvements in gender parity may be reached by focusing on education in the least developed nations, and by focusing on preventative health care, for example in regard to abuse of drugs and alcohol, in medium and highly developed nations.

Please note that more information about the BIGI scale can be found via its dedicated website http://bigi.genderequality.info.

## Supporting information

S1 Table Basic Index of Gender Inequality (BIGI). Note that countries are ranked by the average of the absolute scores (average absolute deviation from parity, AADP) on the three components 1) educational opportunities, 2) healthy life span, and 3) overall life satisfaction. BIGI scores below zero indicate a net advantage for women (bold), and scores above zero an advantage for men (italic). The BIGI score is the average of the ratios *edu* (educational opportunities before age 18), *hls* (healthy life span), and *ols* (overall life satisfaction). These data reflect the five year period 2012–2016. The Human Development Index averaged over the years 2012 to 2015 is given for comparison only.
(DOCX)

## References

[1] Guiso L, Monte F, Sapienza P, Zingales L. Diversity: Culture, gender, and math. Science. 2008;320: 1164–1165. 10.1126/science.1154094 18511674

[2] Stoet G, Geary DC. Sex differences in academic achievement are not related to political, economic, or social equality. Intelligence. 2015;48: 137–151. 10.1016/j.intell.2014.11.006

[3] Benatar D. The Second Sexism: Discrimination Against Men and Boys. London,UK: Wiley-Blackwell; 2012.

[4] World Economic Forum. Global Gender Gap Report 2016 Geneva, Switzerland: World Economic Forum;

[5] Star, S. B. Estimating Gender Disparities in Federal Criminal Cases [Internet]. University of Michigan; 2012. Report No.: 12–018. Available: https://ssrn.com/abstract=2144002

[6] Walmsley R. World Prison Population List (11th Edition) [Internet] London, UK: Institute for Criminal Policy Research at Birbeck University; Available: http://www.prisonstudies.org

[7] Butler P. Number of rough sleepers in England rises for sixth successive year. In: the Guardian [Internet]. 25 Jan 2017 [cited 5 Mar 2018]. Available: http://www.theguardian.com/society/2017/jan/25/number-of-rough-sleepers-in-england-rises-for-sixth-successive-year

[8] Brady KT, Randall CL. Gender differences in substance use disorders. Psychiatr Clin North Am. 1999;22: 241–252. 10385931 10.1016/s0193-953x(05)70074-5

[9] Woodbury MA, Manton KG, Blazer D. Trends in US suicide mortality rates 1968 to 1982: race and sex differences in age, period and cohort components. Int J Epidemiol. 1988;17: 356–362. 3403131 10.1093/ije/17.2.356

[10] Knestaut A. Compensation and Working Conditions Bureau of Labor Statistics; 1996.

[11] Ammermueller A. Violence in European schools: A widespread phenomenon that matters for educational production. Labour Economics. 2012;19: 908–922. 10.1016/j.labeco.2012.08.010

[12] Akila R, Stollery BT, Riihimäki V. Decrements in cognitive performance in metal inert gas welders exposed to aluminium. Occup Environ Med. 1999;56: 632–639. 10615297 10.1136/oem.56.9.632 PMC1757790

[13] OECD. Pensions at a glance. Paris, France: OECD Publishing; 2015.

[14] Omariba DWR, Boyle MH. Family Structure and Child Mortality in Sub-Saharan Africa: Cross-National Effects of Polygyny. Journal of Marriage and Family. 2007;69: 528–543. 10.1111/j.1741-3737.2007.00381.x

[15] Benion B., Joffe L. F., The polygamy question Logan, UT: Utah State University Press; 2016.

[16] Pinker S. The Sexual Paradox: Extreme men, gifted women and the real gender gap Toronto, Canada: Vintage Canada;

[17] Lubinski D, Benbow CP, Kell HJ. Life Paths and Accomplishments of Mathematically Precocious Males and Females Four Decades Later. Psychol Sci. 2014;25: 2217–2232. 10.1177/0956797614551371 25384550

[18] Hakim C. Women, careers, and work-life preferences. British Journal of Guidance & Counselling. 2006;34: 279–294. 10.1080/03069880600769118

[19] Benatar D. Better Never to Have Been: The Harm of Coming into Existence. Oxford: Clarondon Press; 2006.

[20] Pittau MG, Zelli R, Gelman A. Economic Disparities and Life Satisfaction in European Regions. Soc Indic Res. 2010;96: 339–361. 10.1007/s11205-009-9481-2

[21] Hawken A, Munck GL. Cross-National Indices with Gender-Differentiated Data: What Do They Measure? How Valid Are They? Soc Indic Res. 2013;111: 801–838. 10.1007/s11205-012-0035-7

[22] Su R, Rounds J, Armstrong PI. Men and things, women and people: A meta-analysis of sex differences in interests. Psychological Bulletin. 2009;135: 859–884. 10.1037/a0017364 19883140

[23] United Nations Development Programme. Human Development Report 2016. New York, NY: United Nations Development Programme; 2016.

[24] Wilsnack RW, Wilsnack SC, Kristjanson AF, Vogeltanz-Holm ND, Gmel G. Gender and alcohol consumption: patterns from the multinational GENACIS project. Addiction. 2009;104: 1487–1500. 10.1111/j.1360-0443.2009.02696.x 19686518 PMC2844334

[25] UNESCO Institute for Statistics. Reducing global poverty through universal primary and secondary education. Montreal, Canada: UNESCO Institute for Statistics; 2017. Report No.: Policy Paper 32/Fact Sheet 44.

[26] R Core Team. R: A language and environment for statistical computing. [Internet]. Vienna, Austria: R Foundation for Statistical Computing; 2018 Available: http://www.r-project.org/

[27] South A. rworldmap : a new R package for mapping global data. The R Journal. 2011;3: 35–43.

[28] Wang H, Dwyer-Lindgren L, Lofgren KT, Rajaratnam JK, Marcus JR, Levin-Rector A, et al Age-specific and sex-specific mortality in 187 countries, 1970–2010: a systematic analysis for the Global Burden of Disease Study 2010. The Lancet. 2012;380: 2071–2094. 10.1016/S0140-6736(12)61719-X 23245603

[29] Desert Environments of Republic of Chad. Combating Desertification in Asia, Africa and the Middle East. London,UK: Springer; 2013 pp. 169–189.

[30] Ansell N. Secondary Education Reform in Lesotho and Zimbabwe and the Needs of Rural Girls: Pronouncements, policy and practice. Comparative Education. 2002;38: 91–112. 10.1080/0305006012013874

[31] Clutton-Brock T., Isvaran K. Sex differences in ageing in natural populations of vertebrates. Proc Biol Sci. 2007;274: 3097–3104. 10.1098/rspb.2007.1138 17939988 PMC2293943

[32] Popova S, Rehm J, Patra J, Zatonski W. Comparing alcohol consumption in central and eastern Europe to other European countries. Alcohol Alcohol. 2007;42: 465–473. 10.1093/alcalc/agl124 17287207

[33] Murphy A, Roberts B, Stickley A, McKee M. Social factors associated with alcohol consumption in the former Soviet Union: a systematic review. Alcohol Alcohol. 2012;47: 711–718. 10.1093/alcalc/ags077 22813540

[34] Klasen S. UNDP’s Gender‐related Measures: Some Conceptual Problems and Possible Solutions. Journal of Human Development. 2006;7: 243–274. 10.1080/14649880600768595

[35] Fisher M. Saudi Arabia’s oppression of women goes way beyond its ban on driving. Washington Post. 28 Oct 2013. Available: https://www.washingtonpost.com/news/worldviews/wp/2013/10/28/saudi-arabias-oppression-of-women-goes-way-beyond-its-ban-on-driving/. Accessed 5 Mar 2018.

[36] Sengupta S. A Saudi Woman Who Got Behind the Wheel and Never Looked Back. The New York Times 16 6 2017 Available: https://www.nytimes.com/2017/06/16/world/middleeast/saudi-womens-rights.html. Accessed 5 Mar 2018.

[37] Spierings N, Smits J, Verloo M. On the Compatibility of Islam and Gender Equality. Soc Indic Res. 2009;90: 503–522. 10.1007/s11205-008-9274-z

[38] International Association for the Evaluation of Educational Achievement (IEA). Trends in International Mathematics and Science Study (TIMSS). Boston, MA: TIMSS & PIRLS International Study Center, Lynch School of Education, Boston College and IEA; 2016.

[39] UNESCO. UNESCO and Education. Paris, France: United Nations Educational, Scientific and Cultural Organization;

