# Supplementary material for: A simplified approach to measuring national gender inequality
Source: PLoS One. 2019 Jan 3;14(1):e0205349. doi: 10.1371/journal.pone.0205349 (PMC6317789; doi:10.1371/journal.pone.0205349)
Supplement: S1 Table — Note that countries are ranked by the average of the absolute scores (average absolute deviation from parity, AADP) on the three components 1) educational opportunities, 2) healthy life span, and 3) overall life satisfaction. BIGI scores below zero indicate a net advantage for women (bold), and scores above zero an advantage for men (italic). The BIGI score is the average of the ratios edu (educational opportunities before age 18), hls (healthy life span), and ols (overall life satisfaction). These data reflect the five year period 2012–2016. The Human Development Index averaged over the years 2012 to 2015 is given for comparison only. (DOCX) [file pone.0205349.s001.docx]

**Table S1**

| *Rank* | *Rank in overall parity* | *country* | *BIGI* | *AADP* | *Basic Education* | *Healthy Life Span* | *Life Satisfaction* | *Human Development Index* |
| --- | --- | --- | --- | --- | --- | --- | --- | --- |
| 1 | 12 | Bahrain | **-0.00794** | 0.00794 | -0.01264 | 0.00000 | -0.01117 | 0.82050 |
| 2 | 24 | Great Britain | **-0.01455** | 0.01567 | -0.01214 | -0.03318 | 0.00169 | 0.90500 |
| 3 | 27 | Netherlands | **-0.01585** | 0.01585 | -0.01109 | -0.02748 | -0.00898 | 0.92300 |
| 4 | 35 | Iceland | **-0.01768** | 0.01681 | -0.01101 | -0.02437 | -0.01506 | 0.91550 |
| 5 | 32 | New Zealand | **-0.01709** | 0.01709 | -0.01033 | -0.02725 | -0.01368 | 0.91150 |
| 6 | 30 | Serbia | **-0.01670** | 0.01851 | -0.00487 | -0.04794 | 0.00273 | 0.77200 |
| 7 | 39 | Norway | **-0.01950** | 0.01865 | -0.00417 | -0.03303 | -0.01875 | 0.94600 |
| 8 | 34 | Ireland | **-0.01744** | 0.02011 | 0.00401 | -0.04635 | -0.00997 | 0.91375 |
| 9 | 40 | Ecuador | **-0.02012** | 0.02012 | -0.00663 | -0.05348 | -0.00025 | 0.73500 |
| 10 | 31 | Lebanon | **-0.01697** | 0.02016 | 0.00479 | -0.04472 | -0.01098 | 0.76375 |
| 11 | 10 | Malta | **-0.00732** | 0.02170 | -0.01064 | -0.03288 | 0.02157 | 0.84600 |
| 12 | 16 | Belgium | **-0.01118** | 0.02190 | 0.01609 | -0.04925 | -0.00038 | 0.89250 |
| 13 | 2 | Israel | *0.00063* | 0.02230 | 0.02111 | -0.03251 | 0.01328 | 0.89575 |
| 14 | 45 | United Arab Emirates | **-0.02244** | 0.02244 | -0.03587 | 0.00000 | -0.03145 | 0.83425 |
| 15 | 26 | Sweden | **-0.01575** | 0.02253 | 0.01017 | -0.03792 | -0.01950 | 0.90800 |
| 16 | 46 | Denmark | **-0.02347** | 0.02347 | -0.03058 | -0.03604 | -0.00380 | 0.92450 |
| 17 | 23 | Canada | **-0.01417** | 0.02380 | 0.01445 | -0.03777 | -0.01919 | 0.91500 |
| 18 | 11 | Switzerland | **-0.00794** | 0.02391 | 0.02404 | -0.04011 | -0.00757 | 0.93675 |
| 19 | 49 | Australia | **-0.02420** | 0.02420 | -0.01385 | -0.04032 | -0.01844 | 0.93625 |
| 20 | 36 | Czech Rep. | **-0.01833** | 0.02466 | 0.00325 | -0.06448 | 0.00625 | 0.87225 |
| 21 | 52 | Cyprus | **-0.02550** | 0.02550 | -0.01317 | -0.03495 | -0.02838 | 0.85225 |
| 22 | 43 | France | **-0.02177** | 0.02567 | -0.00430 | -0.06686 | 0.00584 | 0.89200 |
| 23 | 4 | Azerbaijan | **-0.00267** | 0.02639 | 0.02781 | -0.04359 | 0.00777 | 0.75350 |
| 24 | 1 | Italy | *0.00021* | 0.02695 | 0.00178 | -0.04011 | 0.03897 | 0.88025 |
| 25 | 87 | Belize | **-0.04794** | 0.02939 | 0.00323 | -0.08355 | -0.00140 | 0.70575 |
| 26 | 20 | Germany | **-0.01299** | 0.02945 | 0.02468 | -0.05147 | -0.01219 | 0.92225 |
| 27 | 58 | Spain | **-0.02990** | 0.02990 | -0.00438 | -0.05832 | -0.02702 | 0.87925 |
| 28 | 21 | Montenegro | **-0.01312** | 0.03023 | 0.03238 | -0.03980 | -0.01852 | 0.80325 |
| 29 | 33 | Austria | **-0.01739** | 0.03057 | 0.01978 | -0.05998 | -0.01196 | 0.89100 |
| 30 | 42 | Luxembourg | **-0.02113** | 0.03091 | -0.03208 | -0.04599 | 0.01467 | 0.89450 |
| 31 | 18 | Albania | **-0.01289** | 0.03223 | 0.02901 | -0.01818 | -0.04950 | 0.76150 |
| 32 | 66 | Malaysia | **-0.03595** | 0.03242 | -0.00505 | -0.05152 | -0.04071 | 0.78450 |
| 33 | 9 | Macedonia | *0.00683* | 0.03244 | 0.02806 | -0.03841 | 0.03085 | 0.74450 |
| 34 | 61 | United States of America | **-0.03294** | 0.03294 | -0.02006 | -0.04757 | -0.03118 | 0.91725 |
| 35 | 62 | Jordan | **-0.03303** | 0.03303 | -0.00468 | -0.02173 | -0.07268 | 0.73900 |
| 36 | 8 | China | *0.00626* | 0.03352 | 0.05967 | -0.03504 | -0.00585 | 0.72700 |
| 37 | 63 | Chile | **-0.03321** | 0.03356 | -0.03905 | -0.06111 | 0.00052 | 0.84100 |
| 38 | 38 | Greece | **-0.01916** | 0.03414 | 0.02247 | -0.04909 | -0.03086 | 0.86325 |
| 39 | 37 | Peru | *0.01863* | 0.03438 | 0.07510 | -0.02362 | 0.00442 | 0.73575 |
| 40 | 60 | Moldova (Republic of) | **-0.03240** | 0.03452 | 0.00318 | -0.09538 | -0.00501 | 0.69550 |
| 41 | 65 | Mexico | **-0.03545** | 0.03545 | -0.04241 | -0.05797 | -0.00595 | 0.75675 |
| 42 | 13 | Madagascar | *0.00997* | 0.03580 | 0.06866 | -0.03652 | -0.00223 | 0.51000 |
| 43 | 68 | Paraguay | **-0.03600** | 0.03600 | -0.05271 | -0.05400 | -0.00128 | 0.68800 |
| 44 | 44 | Slovenia | **-0.02206** | 0.03621 | -0.01060 | -0.07680 | 0.02122 | 0.88600 |
| 45 | 70 | Brazil | **-0.03678** | 0.03678 | -0.03711 | -0.06858 | -0.00464 | 0.74725 |
| 46 | 50 | Romania | **-0.02455** | 0.03692 | -0.01062 | -0.08159 | 0.01856 | 0.79775 |
| 47 | 59 | Kuwait | **-0.03106** | 0.03694 | -0.04861 | 0.00882 | -0.05339 | 0.79550 |
| 48 | 6 | Singapore | *0.00315* | 0.03701 | 0.05710 | -0.04451 | -0.00942 | 0.92275 |
| 49 | 5 | Indonesia | **-0.00309** | 0.03777 | 0.05202 | -0.03468 | -0.02660 | 0.68350 |
| 50 | 72 | Finland | **-0.03802** | 0.03802 | -0.00628 | -0.06502 | -0.04276 | 0.89125 |
| 51 | 25 | Kyrgyz Republic | **-0.01509** | 0.03857 | 0.02218 | -0.08049 | 0.01304 | 0.65725 |
| 52 | 29 | Georgia | **-0.01654** | 0.03866 | 0.03072 | -0.08279 | 0.00246 | 0.76275 |
| 53 | 73 | Bangladesh | **-0.03892** | 0.03892 | -0.03523 | -0.00904 | -0.07251 | 0.57225 |
| 54 | 41 | Slovak Republic | **-0.02104** | 0.03991 | 0.00314 | -0.09143 | 0.02516 | 0.84150 |
| 55 | 76 | Tajikistan | *0.03958* | 0.04158 | 0.10556 | -0.00299 | 0.01618 | 0.62275 |
| 56 | 22 | Bosnia and Herzegovina | **-0.01387** | 0.04215 | 0.03922 | -0.05714 | -0.03010 | 0.74350 |
| 57 | 47 | Hungary | **-0.02361** | 0.04249 | 0.01302 | -0.09915 | 0.01529 | 0.83200 |
| 58 | 80 | Japan | **-0.04286** | 0.04286 | -0.01000 | -0.06940 | -0.04917 | 0.89950 |
| 59 | 19 | Bulgaria | **-0.01290** | 0.04362 | 0.02541 | -0.08478 | 0.02068 | 0.78850 |
| 60 | 81 | Costa Rica | **-0.04373** | 0.04373 | -0.04921 | -0.04225 | -0.03972 | 0.77025 |
| 61 | 67 | Kazakhstan | **-0.03598** | 0.04513 | 0.00042 | -0.12167 | 0.01330 | 0.78950 |
| 62 | 56 | Croatia | **-0.02832** | 0.04519 | -0.04455 | -0.06571 | 0.02531 | 0.82175 |
| 63 | 85 | Honduras | **-0.04623** | 0.04623 | -0.08593 | -0.04644 | -0.00631 | 0.62000 |
| 64 | 78 | Korea (Republic of) | **-0.04113** | 0.04664 | 0.00827 | -0.07243 | -0.05922 | 0.89675 |
| 65 | 82 | Latvia | **-0.04533** | 0.04759 | -0.01947 | -0.11991 | 0.00338 | 0.82350 |
| 66 | 86 | Estonia | **-0.04631** | 0.04791 | -0.01740 | -0.12394 | 0.00240 | 0.86100 |
| 67 | 54 | El Salvador | **-0.02645** | 0.04903 | 0.03387 | -0.08932 | -0.02390 | 0.67725 |
| 68 | 89 | Panama | **-0.04907** | 0.04907 | -0.07370 | -0.05781 | -0.01569 | 0.78150 |
| 69 | 28 | Bolivia (Plurinational State of) | *0.01648* | 0.04956 | 0.06422 | -0.04963 | 0.03483 | 0.66800 |
| 70 | 14 | Zimbabwe | **-0.01028** | 0.04996 | 0.06027 | -0.07089 | -0.01872 | 0.50225 |
| 71 | 91 | Poland | **-0.05090** | 0.05090 | -0.01735 | -0.09907 | -0.03628 | 0.84875 |
| 72 | 3 | Saudi Arabia | **-0.00155** | 0.05144 | 0.07483 | -0.03087 | -0.04862 | 0.84075 |
| 73 | 53 | Viet Nam | **-0.02598** | 0.05251 | 0.03979 | -0.09025 | -0.02748 | 0.67600 |
| 74 | 75 | Botswana | **-0.03953** | 0.05468 | -0.13115 | -0.01016 | 0.02272 | 0.69650 |
| 75 | 77 | Lithuania | **-0.03962** | 0.05484 | 0.01067 | -0.14168 | 0.01217 | 0.84225 |
| 76 | 69 | Rwanda | *0.03671* | 0.05536 | 0.09620 | -0.02934 | 0.04055 | 0.49100 |
| 77 | 97 | Colombia | **-0.05558** | 0.05558 | -0.07398 | -0.06360 | -0.02917 | 0.72075 |
| 78 | 48 | Mauritius | **-0.02381** | 0.05565 | 0.05216 | -0.07756 | -0.03725 | 0.77350 |
| 79 | 55 | Burundi | *0.02732* | 0.05646 | 0.12470 | -0.03787 | -0.00681 | 0.40300 |
| 80 | 15 | Kenya | *0.01113* | 0.05649 | 0.10143 | -0.03056 | -0.03748 | 0.54800 |
| 81 | 100 | Sri Lanka | **-0.05761** | 0.05650 | -0.05267 | -0.06873 | -0.04809 | 0.76175 |
| 82 | 98 | South Africa | **-0.05560** | 0.05654 | -0.10774 | -0.06047 | 0.00141 | 0.66075 |
| 83 | 7 | Turkey | **-0.00616** | 0.05673 | 0.07585 | -0.05373 | -0.04059 | 0.76100 |
| 84 | 99 | Portugal | **-0.05676** | 0.05676 | -0.08245 | -0.06027 | -0.02754 | 0.83700 |
| 85 | 101 | Jamaica | **-0.05821** | 0.05908 | -0.09979 | -0.06667 | -0.01078 | 0.72825 |
| 86 | 104 | Argentina | **-0.05960** | 0.05960 | -0.08308 | -0.07246 | -0.02325 | 0.82525 |
| 87 | 105 | Thailand | **-0.06116** | 0.06116 | -0.07061 | -0.08641 | -0.02645 | 0.73700 |
| 88 | 83 | Belarus | **-0.04567** | 0.06261 | -0.01056 | -0.15196 | 0.02530 | 0.79650 |
| 89 | 74 | Russia | **-0.03949** | 0.06416 | 0.01739 | -0.15548 | 0.01963 | 0.80275 |
| 90 | 88 | Trinidad and Tobago | **-0.04859** | 0.06463 | -0.05030 | -0.03800 | -0.10558 | 0.77750 |
| 91 | 106 | Armenia | **-0.06523** | 0.06523 | -0.10006 | -0.08600 | -0.00962 | 0.73975 |
| 92 | 107 | Dominican Rep. | **-0.06627** | 0.06627 | -0.12793 | -0.03097 | -0.03991 | 0.71525 |
| 93 | 103 | Qatar | **-0.05937** | 0.06628 | -0.10421 | 0.02353 | -0.07111 | 0.85200 |
| 94 | 108 | Venezuela (Bolivarian Republic of) | **-0.06653** | 0.06653 | -0.09542 | -0.07570 | -0.02848 | 0.76925 |
| 95 | 109 | Nicaragua | **-0.06813** | 0.06813 | -0.13631 | -0.05758 | -0.01050 | 0.63825 |
| 96 | 71 | Syria | *0.03680* | 0.06987 | 0.12976 | -0.06117 | 0.01868 | 0.57475 |
| 97 | 51 | Ukraine | **-0.02481** | 0.07017 | -0.01458 | -0.12789 | 0.06805 | 0.74525 |
| 98 | 92 | Tanzania (United Republic of) | *0.05197* | 0.07155 | 0.15517 | -0.02937 | 0.03010 | 0.51875 |
| 99 | 96 | Ghana | *0.05442* | 0.07192 | 0.15320 | -0.02625 | 0.03631 | 0.57500 |
| 100 | 111 | Uruguay | **-0.07366** | 0.07366 | -0.10006 | -0.07714 | -0.04378 | 0.79200 |
| 101 | 17 | Guatemala | *0.01220* | 0.07688 | 0.13361 | -0.07196 | -0.02505 | 0.62550 |
| 102 | 112 | Mongolia | **-0.07693** | 0.07693 | -0.06632 | -0.11113 | -0.05333 | 0.72925 |
| 103 | 93 | Tunisia | *0.05382* | 0.07701 | 0.18283 | -0.03936 | 0.00885 | 0.72250 |
| 104 | 79 | Algeria | *0.04284* | 0.08338 | 0.18834 | -0.01587 | -0.04591 | 0.74150 |
| 105 | 116 | Suriname | **-0.08442** | 0.08552 | -0.17081 | -0.08162 | 0.00412 | 0.72225 |
| 106 | 84 | Uganda | *0.04568* | 0.08607 | 0.19763 | -0.05435 | -0.00624 | 0.48550 |
| 107 | 110 | Malawi | *0.07135* | 0.09029 | 0.22548 | -0.02840 | 0.01698 | 0.46850 |
| 108 | 90 | Cambodia | *0.04941* | 0.09393 | 0.18443 | -0.06679 | 0.03058 | 0.55500 |
| 109 | 57 | Iran (Islamic Republic of) | **-0.02898** | 0.09678 | 0.10170 | -0.03137 | -0.15729 | 0.77175 |
| 110 | 94 | Cameroon | *0.05383* | 0.09809 | 0.22685 | -0.01020 | -0.05722 | 0.51000 |
| 111 | 64 | Egypt | *0.03333* | 0.09841 | 0.19761 | -0.04158 | -0.05604 | 0.68650 |
| 112 | 121 | Philippines | **-0.09874** | 0.09874 | -0.15951 | -0.08839 | -0.04832 | 0.67700 |
| 113 | 115 | Zambia | *0.08292* | 0.10469 | 0.25035 | -0.03230 | 0.03142 | 0.57250 |
| 114 | 117 | India | *0.08477* | 0.10761 | 0.28583 | -0.03425 | 0.00275 | 0.61125 |
| 115 | 120 | Nigeria | *0.09076* | 0.11014 | 0.30240 | -0.00426 | -0.02377 | 0.52175 |
| 116 | 113 | Angola | *0.07718* | 0.11298 | 0.27046 | -0.05968 | -0.00879 | 0.52850 |
| 117 | 122 | Bhutan | *0.10976* | 0.11433 | 0.31798 | -0.02150 | 0.00352 | 0.59900 |
| 118 | 119 | Mauritania | *0.09051* | 0.12053 | 0.29360 | -0.04503 | 0.02294 | 0.50900 |
| 119 | 102 | Namibia | **-0.05866** | 0.12256 | -0.21754 | -0.04777 | 0.10235 | 0.63350 |
| 120 | 95 | Morocco | *0.05386* | 0.12388 | 0.26661 | -0.02888 | -0.07616 | 0.64150 |
| 121 | 114 | Nepal | *0.08147* | 0.13153 | 0.31949 | -0.02000 | -0.05509 | 0.55225 |
| 122 | 123 | Senegal | *0.11005* | 0.13227 | 0.36348 | -0.02967 | -0.00366 | 0.48550 |
| 123 | 127 | Burkina Faso | *0.12362* | 0.13767 | 0.39169 | -0.02107 | 0.00024 | 0.39750 |
| 124 | 124 | Pakistan | *0.11617* | 0.14280 | 0.38846 | -0.00338 | -0.03656 | 0.54450 |
| 125 | 125 | Ethiopia | *0.11647* | 0.14572 | 0.37882 | -0.04388 | 0.01447 | 0.43775 |
| 126 | 128 | Mozambique | *0.12896* | 0.15070 | 0.41463 | -0.01286 | 0.02461 | 0.41150 |
| 127 | 129 | Lesotho | **-0.15264** | 0.15520 | -0.36738 | -0.06563 | -0.03260 | 0.49175 |
| 128 | 126 | Cote d'Ivoire | *0.11995* | 0.15663 | 0.41494 | -0.03804 | -0.01689 | 0.46275 |
| 129 | 132 | Mali | *0.16047* | 0.16047 | 0.45581 | 0.00148 | 0.02414 | 0.43275 |
| 130 | 118 | Yemen | *0.09023* | 0.16653 | 0.38513 | -0.02546 | -0.08900 | 0.49475 |
| 131 | 130 | Guinea | *0.15346* | 0.17123 | 0.48838 | -0.02000 | -0.00532 | 0.41150 |
| 132 | 131 | Liberia | *0.15764* | 0.17410 | 0.49762 | -0.01887 | -0.00582 | 0.42450 |
| 133 | 133 | Benin | *0.18726* | 0.18961 | 0.49987 | -0.00980 | 0.05915 | 0.47675 |
| 134 | 134 | Chad | *0.23114* | 0.24009 | 0.66015 | -0.01343 | 0.04670 | 0.39175 |
